# Insulin resistance, aging biology, and non- communicable chronic diseases: a narrative review of bidirectional mechanisms and translational implications

**DOI:** 10.3389/fendo.2026.1891078

**Published:** 2026-07-27

**Authors:** Sidhartha Gautam Senapati

**Affiliations:** Department of Internal Medicine, Texas Tech University Health Sciences Center El Paso, El Paso, TX, United States

**Keywords:** aging, Alzheimer’s disease, cardiovascular disease, frailty, hyperinsulinemia, inflammaging, insulin resistance, metabolic syndrome

## Abstract

**Background:**

Insulin resistance has been considered a metabolic disorder related to obesity, metabolic syndrome, and type 2 diabetes mellitus. Growing evidence points to possible interactions between insulin resistance and hyperinsulinemia and the biological aging process and age-related non-communicable diseases, like cardiovascular disease, neurodegenerative disorders, sarcopenia, frailty, adipose tissue dysfunction, chronic kidney disease, and liver disease. Most published associations lack causality, and some biological aging mechanisms may also independently increase the risk for both insulin resistance and chronic disease.

**Aim:**

In this narrative review, we summarize bidirectional connections between insulin resistance, compensatory hyperinsulinemia, aging biology, and age-related non-communicable diseases and the quality of existing data.

**Methods:**

We performed a structured narrative literature review for mechanistic, translational, omics, epidemiologic, and intervention studies on the connection between insulin resistance and biological mechanisms of aging and chronic disease.

**Results:**

Mechanisms of age-related disease that may be affected by insulin resistance include insulin/IGF-1 signaling disruption, hyperinsulinemia, mitochondria dysfunction, oxidative stress, endothelial dysfunction, adipokine imbalance, chronic low-grade inflammation, cell senescence, ectopic lipids accumulation, AGE-RAGE signaling, and autophagy impairment. Aging mechanisms, such as cell senescence, mitochondria dysfunction, inflammaging, altered nutrient sensing, impaired proteostasis, adipose tissue remodeling, and physical inactivity may contribute to insulin resistance. Quality of evidence differs from strong to associative and exploratory depending on disease domain.

**Conclusion:**

It is important to understand insulin resistance as an important mediator in reciprocal network of connections between metabolism, biological aging, and age-related chronic diseases, rather than one of the causes of aging.

## Introduction and definition of insulin resistance

Insulin resistance (IR) denotes a basic pathophysiologic state associated with an insufficient response of target tissue to a particular amount of insulin. Insulin resistance has been recognized for nearly a century, it was not until recently that insulin resistance was regarded as a crucial link between aging and developing a disease. Despite the fact that it is the key characteristic of type 2 diabetes, its importance does not reduce to glucose metabolism and involves several organs and systems. It may represent an important metabolic mediator linking obesity, lifestyle-related metabolic stress, biological aging pathways, and several age-related chronic diseases (1). The prevalence of insulin resistance has increased across age groups, largely paralleling rising obesity, sedentary behavior, and dietary changes (2).

**Figure 1:**
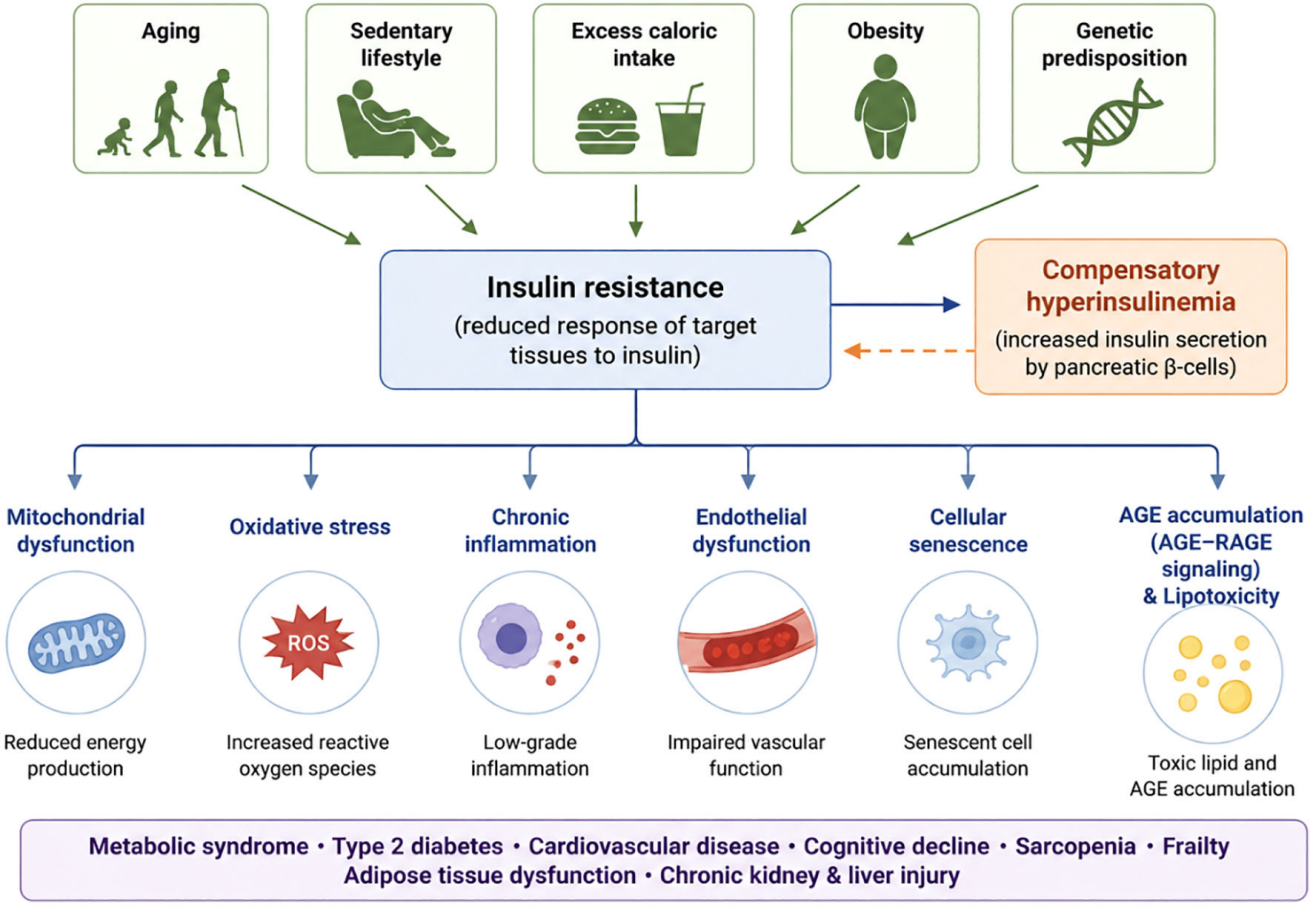
The conceptual relationship between insulin resistance, hyperinsulinemia, aging, and chronic diseases. Insulin resistance and hyperinsulinemia can be caused by such factors as aging, sedentary lifestyle, overconsumption of calories, obesity, and genetic predispositions. This can also have some relation to mitochondrial dysfunction, oxidative stress, inflammation, endothelial dysfunction, cellular senescence, AGE-RAGE mechanism, and lipotoxicity, which can lead to chronic diseases in elderly patients. The above-described relationship is bi-directional and cannot be treated as an example of linear cause-and-effect relationship.

The syndrome of insulin resistance, as suggested by Gerald Reaven, includes metabolic disorders such as dyslipidemia, high blood pressure, being overweight, and glucose intolerance. Insulin resistance is generally accompanied by hyperinsulinemia as a consequence of increased insulin secretion from the beta cells of the pancreas to preserve glucose balance. But it is important to note that this relationship works in both directions depending on the stage: in most cases, insulin resistance occurs before hyperinsulinemia, and vice versa (3, 4). This reciprocal relationship has important therapeutic implications. Insulin resistance and hyperinsulinemia may be thought of as an essential biological nexus between aging, metabolic stress due to lifestyle, obesity, and genetic risk factors that lead to various other chronic conditions. Insulin resistance may participate in reciprocal biological pathways involving mitochondrial dysfunction, oxidative stress, inflammation, endothelial dysfunction, cellular senescence, AGE-RAGE signaling, and lipotoxicity. These interconnected pathways may contribute to the development or progression of metabolic syndrome, type 2 diabetes, cardiovascular disorders, neuropathy, sarcopenia, frailty, adipocyte dysfunction, and chronic kidney and liver disease (**Figure 1**).

Recent developments in transcriptomic profiling, single-cell RNA sequencing, spatial transcriptomics, and bioinformatic integration have enhanced the knowledge about the role of aging on insulin resistance beyond systemic models alone. The results reveal that aging-related metabolic abnormalities involve not only fat cell enlargement and obesity but also alterations in adipose progenitors, stromal compartments, inflammatory responses, endothelial changes, extracellular matrix modifications, and intercellular signaling (5–9). Hence, in this review, the classic ideas related to metabolic function will be considered along with more modern views to highlight how insulin resistance acts as a two-way street in aging and chronic illness.

While the focus in this review is on the associations between aging and diseases through mechanisms such as insulin resistance and hyperinsulinemia, one must not consider this causality model to be a universal explanation that encompasses all aspects of pathology due to aging. The connection between aging and insulin resistance is highly multifactorial, reciprocal, and variable. For some individuals, insulin resistance will be triggered by prior hyperinsulinemia; in other cases, the onset of insulin resistance will be caused by β-cell malfunction, chronic inflammation, aging mitochondria, dysfunctional adipose tissue, epigenetics, or metabolism due to lifestyle choices.

## Methods

This narrative review aims to synthesize and interpret current evidence connecting insulin resistance, hyperinsulinemia, biological aging, and age-related non-communicable chronic diseases. For this purpose, we performed a systematic literature search in PubMed, Scopus, and Web of Science. Search keywords combined the following terms: “insulin resistance,” “hyperinsulinemia,” “aging,” “biological aging,” “non-communicable disease,” “metabolic syndrome,” “type 2 diabetes,” “cardiovascular disease,” “Alzheimer’s disease,” “neurodegeneration,” “sarcopenia,” “frailty,” “adipose tissue aging,” “cellular senescence,” “mitochondrial dysfunction,” “oxidative stress,” “inflammaging,” “impaired proteostasis,” “autophagy,” “advanced glycation end products,” “lipotoxicity,” “single-cell RNA sequencing,” “spatial transcriptomics,” “transcriptomics,” “metformin,” “GLP-1 receptor agonists,” “exercise,” “caloric restriction,” and “senolytics”.

The literature search spanned from the earliest records available to June 2026 with particular focus on articles published in the last five years. The earlier landmark studies were considered only if they laid out the conceptual foundations for insulin signaling, metabolic syndrome, hyperinsulinemia, aging biology, or age-related disease pathogenesis. After title/abstract evaluation for relevance, full-text examination was performed for articles investigating associations, translations, epidemiology, omics approaches, or interventions in relation to insulin resistance and age-related effects.

For evidence synthesis, priority was assigned to the following types of studies: randomized controlled trials and interventional studies in humans; longitudinal cohort studies; mechanistic and translational studies in humans; omics studies with human samples; animal studies; *in vitro* studies; and high-quality narrative reviews or systematic reviews. Animal and *in vitro* studies were considered mechanistic or generating hypotheses, unless there was an evidence base in humans. Articles unrelated to insulin resistance, hyperinsulinemia, biological aging, or age-related non-communicable diseases were excluded. Additionally, any paper lacking the full text was not considered.

Since this is a narrative review rather than a systematic review or meta-analysis, PRISMA screening, statistical synthesis, and risk-of-bias assessment were not performed. In order to minimize bias and enhance interpretability of the evidence, the results are presented in a thematic way and classified as associative, mechanistic, tissue-specific, omics, interventional, or unresolved. In the case of unclear causation, the manuscript clearly separates association, biological plausibility, and interventional evidence.

## The insulin resistance-aging nexus

### Insulin signaling and longevity

Insulin signaling and aging can be regarded as one of the most evolutionarily conserved biological relationships observed throughout different living creatures (10). Multiple experiments conducted on various animal models such as nematodes, fruit flies, or mice have shown that decreased insulin level or decreased activity of insulin signaling contribute to enhanced longevity and delay the onset of the physiological consequences of aging (10). On the contrary, in humans, increased insulin secretion and subsequent insulin resistance correlate with higher probability of developing age-related illnesses and decreased lifespan of people, which means that increased concentration of insulin may contribute to the aging process itself (10).

In terms of mechanisms, this relationship is mediated by significant role of insulin in regulation of anabolic-catabolic processes. Increased insulin concentrations lead to the enhanced lipogenesis and fatty acid deposition, increased synthesis of proteins, and deposition of proteins with reduced or no functionality due to the low rate of protein catabolism (10). Simultaneously, high concentration of insulin inhibits autophagic activity and leads to impaired function of endothelial nitric oxide synthase, resulting in dysfunctional blood vessels (10). The stress state caused by anabolic overstimulation activates various protective factors such as Nrf2, AMP-activated kinase, and unfolded protein response (10). However, the older a cell or organism becomes, the less resistant it is to the stress induced by insulin; therefore, this mechanism results in a positive feedback loop contributing to the aging process (10).

### Hyperinsulinemia as a potential primary event

Recently, many publications have disputed the dogma that hyperinsulinemia is merely an adaptive mechanism to overcome insulin resistance. According to this hypothesis, hyperinsulinemia in certain conditions could act as a causative factor that promotes the development of insulin resistance by decreasing insulin receptor responsiveness, altering pulsatile release of insulin, increasing deposition of lipids, inhibiting lipolysis, and generating metabolic stress (4, 10, 11). Moreover, hyperinsulinemia could play an important role in inducing cell senescence, since experimental data indicate that insulin-induced cell senescence is mediated via insulin receptor-dependent p53 and p21 signaling pathways (11).

However, this hypothesis continues to develop and cannot be considered as a proven cause-and-effect relationship. Insulin resistance often develops secondary to inflammation, ectopic fat deposition, impaired mitochondria, sedentary lifestyle, age-associated changes in adipose tissue distribution, and intrinsic beta cell dysfunction. Importantly, the progression of beta cell dysfunction leads to the development of overt type 2 diabetes in patients with compensated insulin resistance. Therefore, hyperinsulinemia might be the cause, consequence, or enhancer of insulin resistance in specific circumstances (12).

## Alternative frameworks and causality

Insulin resistance and aging should be viewed in relation to several alternative frameworks. For example, according to the first framework, known as the metabolic stress model, chronic exposure to nutrient overload, ectopic fat deposition, and hyperinsulinemia can lead to oxidative stress, mitochondrial dysfunction, decreased autophagy, and cellular senescence. The second one, which is called inflammation-first model, highlights chronic low-grade inflammation, immune cells’ recruitment, and cytokines’ signaling as potential mechanisms affecting the functioning of insulin receptor substrates as well as downstream PI3K-Akt pathway. The third proposed model, which is related to mitochondria, postulates reduced mitochondria’s capacity for oxidation with higher ROS production as a possible mechanism leading to decreased metabolic flexibility and sensitivity to insulin. The fourth proposed model, or the β-cell dysfunction model, focuses on impaired β-cells’ function, β-cells’ dedifferentiation, and their progressive senescence and glucolipotoxicity (12). Finally, the epigenetics model of aging suggests that aging may affect metabolic tissue function via age-related changes in DNA methylation, chromatin remodeling, and gene expression regulation.

All above models are not mutually exclusive. Aging-related insulin resistance is thought to result from interactions between metabolic stress, chronic inflammation, mitochondria-related dysfunction, impairment in insulin secretion and β-cell dedifferentiation and/or senescence, as well as β-cell failure. Moreover, epigenetics is considered to play a role in the development of this phenomenon. Hence, any claim about insulin resistance’s driving role in aging processes should be made carefully. To clarify the strength of evidence supporting different claims in this review, **Table 1** summarizes associative, mechanistic, tissue-specific, omics-based, interventional, and unresolved evidence categories linking insulin resistance, aging, and chronic disease.

**Table 1:** Evidence categories linking insulin resistance, aging, and chronic disease.

Evidence type	Examples	Interpretation
Associative evidence	Cohort studies linking HOMA-IR, fasting insulin, metabolic syndrome, obesity, or diabetes with cardiovascular disease, dementia, frailty, and mortality	Supports clinical correlation but cannot establish causality
Mechanistic evidence	Studies showing mitochondrial dysfunction, oxidative stress, inflammation, senescence, lipotoxicity, and impaired insulin signaling in metabolic tissues	Supports biological plausibility
Tissue-specific evidence	Studies of skeletal muscle, liver, adipose tissue, brain, vascular tissue, and β-cells	Demonstrates that insulin resistance differs across organs
Omics evidence	Single-cell RNA sequencing, spatial transcriptomics, adipose progenitor studies, and biomarker discovery studies	Identifies emerging cell states and molecular regulators requiring validation
Interventional evidence	Exercise, caloric restriction, weight loss, metformin, GLP-1 receptor agonists, senolytic studies	Provides stronger causal inference when interventions improve both insulin resistance and clinical outcomes
Unresolved/debated areas	Hyperinsulinemia as cause versus consequence of insulin resistance; insulin resistance as driver versus marker of aging	Requires longitudinal and experimental confirmation

Evidence types relating insulin resistance, aging, and chronic diseases. The purpose of **Table 1** is to classify different types of evidence that help determine how strong some claims have been in the literature review.

Though insulin resistance is closely linked to several aging-associated phenotypes, these relationships should not be viewed as uniformly indicative of causation. Mechanisms of aging like mitochondrial malfunctioning, inflammation, cellular senescence, disruption of proteostasis, autophagic dysregulation, epigenomic alterations, adipose tissue dysfunction, and lower physical activity levels can act independently of each other and contribute to insulin resistance as well as aging-related disease. Hence, insulin resistance should be seen not as an originator of the aging process but as a context-specific mediator and amplification mechanism that exists in reciprocal biological circuits. There is different evidence strength in various diseases; human studies involving intervention and longitudinal observation are strong for cardiometabolic diseases while less direct or mechanistic evidence exists in neurodegenerative diseases, sarcopenia, frailty, and omics at tissue level.

## Bidirectional mechanisms that connect aging with insulin resistance

Aging and insulin resistance have a reciprocal relationship whereby insulin resistance accelerates aging-related changes while aging promotes insulin resistance through multiple biological mechanisms. Biological mechanisms that promote insulin resistance include cellular senescence, mitochondrial dysfunction, oxidative stress, chronic inflammation, impaired proteostasis, altered autophagy, and dysfunctional nutrient sensing (2, 10, 13–16). During aging, cells accumulate in the senescence state that secretes a complex mixture of pro-inflammatory cytokines, matrix remodeling enzymes, growth factors, and chemoattractants collectively known as the senescence associated secretory phenotype (16). This process interferes with insulin signaling by activating inflammatory kinases and inhibiting insulin receptor substrate signaling to suppress PI3K/Akt signaling and glucose transporter translocation (14, 16, 17).

Another important pathway that connects aging with insulin resistance is mitochondrial dysfunction. Aging is marked by dysfunctional mitochondrial biogenesis, dynamics, bioenergetics, and oxidative stress through leakage of electrons during oxidative phosphorylation (13, 18). Oxidative stress impairs insulin action through two mechanisms: direct inhibition of signaling processes through protein oxidation and activation of stress and inflammatory pathways that inhibit signaling processes (13, 18). Other biological mechanisms that contribute to this process are endoplasmic reticulum stress and dysfunctional proteostasis that induce unfolded protein response and inflammatory responses to suppress metabolic pathways (14, 16).

Insulin resistance accelerates aging-related changes through altered nutrient sensing pathways. Over activation of mTOR signaling, impaired AMPK activity, sirtuin signaling, and dysfunctional insulin/IGF-1 signaling increase anabolic pressure and impair autophagy leading to oxidative stress and metabolic inflexibility (10, 13, 16). In summary, these alterations make cells less flexible to changing levels of nutrients and metabolic pressure.

On the other hand, insulin resistance can accelerate some biological processes involved in aging. For example, hyperinsulinemia creates anabolic pressure that increases lipid deposition and oxidative stress. It also inhibits autophagy and induces senescence signaling pathway (4, 10, 11, 16). For example, hepatocyte senescence is mediated by insulin receptor-dependent activation of p53 and p21 signaling during chronic hyperinsulinemia (11). Consequently, insulin resistance accelerates aging through endothelial dysfunction, lipotoxicity, impaired mitochondrial quality control, inflammation, and metabolic inflexibility (1, 10, 13, 19). Therefore, there is a feedback relationship between aging and insulin resistance.

## Insulin resistance and metabolic syndrome: the clinical nexus

### Definition and epidemiology of metabolic syndrome

Metabolic syndrome represents a clinical phenotype in which insulin resistance interacts with abdominal adiposity, dyslipidemia, elevated blood pressure, chronic low-grade inflammation, and impaired glucose metabolism. Rather than functioning as a single disease entity, metabolic syndrome reflects a high-risk biological state in which insulin resistance amplifies cardiometabolic vulnerability across multiple organs. In aging, this risk is further intensified by visceral fat redistribution, reduced adipose tissue expandability, adipokine imbalance, physical inactivity, and inflammaging. Thus, metabolic syndrome provides one of the clearest clinical examples of insulin resistance acting as a disease amplifier rather than as an isolated causal pathway (20–23).

## Cellular and molecular mechanisms linking insulin resistance to pathology

### Mitochondrial dysfunction and oxidative stress

In this respect, mitochondrial dysfunction and oxidative stress can be seen as key associations of insulin resistance with pathological features related to aging. Mitochondrial dysfunctions may lead to reduced metabolic plasticity, increased production of reactive oxygen species, and problems in insulin signaling pathways. On the other hand, insulin resistance and nutrient overload may aggravate mitochondrial stress via lipid overload, inflammation, and dysfunctional mitochondrial quality control. The interactions between mitochondrial dysfunction and insulin resistance are thus likely to be two-way (13, 18).

### Chronic inflammation and immunosenescence

Chronic inflammation is an important risk factor for the process of aging and associated age-related diseases, which is directly related to insulin resistance and metabolism (14). Chronic inflammation and insulin resistance interact through several pathways: chronic inflammation inhibits lipid storage and adipocyte functions, results in mitochondrial dysfunction and endoplasmic reticulum stress, and affects insulin signal transduction pathways in various tissues (14). Furthermore, insulin resistance stimulates chronic inflammation through several mechanisms, thus creating a cycle of increasing inflammation and metabolic dysfunction (14).

With aging, immune cells penetrate into adipose and other metabolic tissues and induce inflammatory processes accompanied by altered phenotypic features and impaired immune cells functioning (23). The presence of senescent immune cells, especially senescent T-cells, leads to the condition of immunosenescence, which is characterized by disrupted immune system activity and increased levels of pro-inflammatory mediators (23). There is evidence that T-cell senescence contributes to the development of inflammation and insulin resistance. In fact, recent findings reveal a strong connection between senescent T cells, inflammation, and insulin resistance, indicating that senescent T cells may play a role in the initiation and development of metabolic disorders due to inflammation-induced insulin resistance (15).

### Advanced glycation end products and lipotoxicity

The next factor that should be considered is the role of advanced glycation end products (AGEs), which are especially important in this context. AGEs are toxic metabolites that tend to accumulate during aging; however, they may be also introduced into the body due to high temperature processing of food products (24). AGEs increase protein, lipid, and nucleotide damage due to direct interaction and activation of AGEs receptor (RAGE) (24). There is evidence showing that dietary AGEs are associated with increased AGE body burden, which is related to insulin resistance (25). It should be noted that reduced consumption of AGEs due to simple changes in cooking style results in improved insulin sensitivity regardless of the energy intake or adiposity level (24). Here, the phrase AGE accumulation means the gradual buildup of advanced glycation end products in tissues and RAGE activation and does not mean “age” which is chronological time. AGE-RAGE signaling induces oxidative stress, activation of nuclear factor-κB, production of inflammatory cytokines, endothelial dysfunction, increased stiffness of the extracellular matrix, and cellular senescence. The above processes can result in poor insulin signaling leading to vascular, adipose, and kidney diseases.

Another mechanism that should be mentioned is lipotoxicity. Lipids and their byproducts accumulate in an excessive amount during aging and metabolic disorders leading to mitochondrial dysfunction, impaired β-oxidation, and increased ROS generation (26). The accumulation of lipids causes several adverse reactions resulting in disruption of the insulin signaling pathway and the release of pro-inflammatory factors promoting sarcopenic obesity development (27). Lipids tend to accumulate within skeletal muscles and livers, forming the vicious circle since fatty liver and fatty muscles activate inflammatory response and increase oxidative stress and mitochondrial dysfunction affecting insulin signaling and causing muscle atrophy (27).

**Figure 2:**
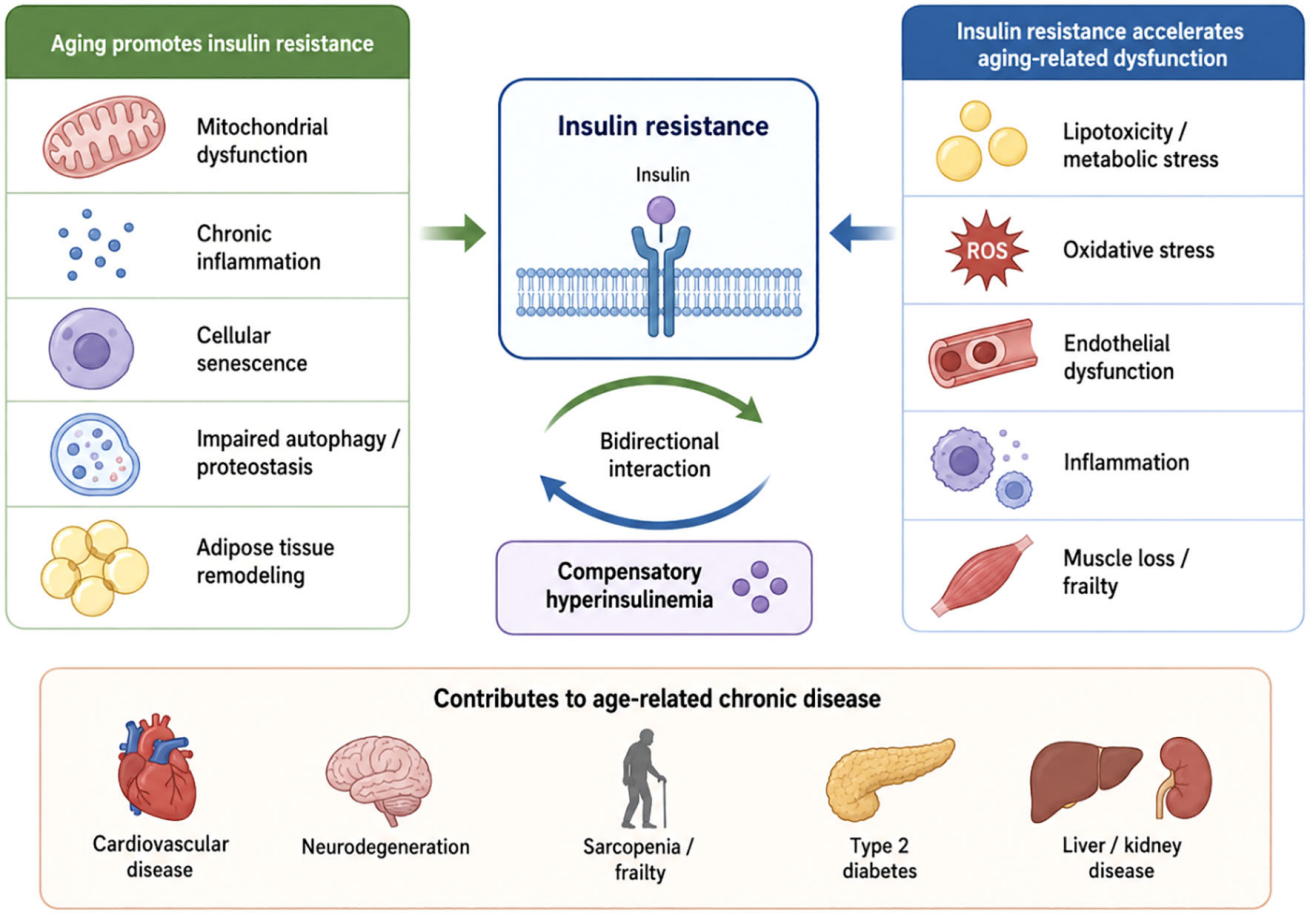
Two-way connection between aging and insulin resistance. Aging might cause insulin resistance via mitochondrial dysfunction, oxidative stress, inflammatory processes, protein homeostasis disruption, alterations in autophagy, imbalances in nutrient sensing, and cell senescence. Insulin resistance and its consequence, hyperinsulinemia, may lead to enhanced tissue dysfunction via metabolic stress, lipotoxicity, endothelial dysfunction, inflammation, and disturbances in mitochondrial quality control. AMPK, AMP-activated protein kinase; DAG, diacylglycerol; eNOS, endothelial nitric oxide synthase; FOXO, forkhead box O; GLUT4, glucose transporter type 4; IGF-1, insulin-like growth factor-1; IL-6, interleukin-6; mTOR, mechanistic target of rapamycin; NO, nitric oxide; PI3K, phosphoinositide 3-kinase; ROS, reactive oxygen species.

The pathological consequences of insulin resistance act through different molecular mechanisms, which reinforce each other. Insulin resistance affects insulin signaling, leading to poor functioning of IRS-1/PI3K/Akt, GLUT4 transport, nitric oxide availability from endothelium, as well as the ratio between mTOR/FOXO. This causes mitochondrial dysfunction, oxidative stress, inflammation, immunosenescence, accumulation of AGEs, lipotoxicity, cell senescence, and defects in autophagy. Interconnection between different mechanisms leads to a vicious circle of metabolic damage and organ dysfunction (**Figure 2**).

## Insulin resistance mechanisms in aging-related tissues

While insulin resistance is a systemic metabolic state, aging affects the cellular and molecular biology of insulin action in a tissue-specific manner. Hence, the need for a tissue-specific approach in examining the interplay between aging and insulin resistance (5–8, 22, 28).

### Adipose tissue

The aged adipose tissue is defined by dysfunctional adipogenesis, decreased adipose storage capacity, increase of visceral fat mass, spillover of ectopic lipids, adipokine dysregulation, fibrosis, recruitment of immune cells into adipose tissue, and accumulation of senescent stromal and adipose progenitor cells (5–8, 16, 22, 28). Loss of subcutaneous adipose tissue expandability makes fatty acid deposition in this tissue unsafe and leads to its redistribution into liver, skeletal muscle, and visceral fats (22, 28). Ectopic deposition of lipids causes lipotoxicity and inflammation in insulin-sensitive tissues. Single-cell and spatial transcriptome analysis showed aging to be linked with remodeling of adipose tissue-specific progenitor, stromal, immune and endothelial cell types (5–8). In addition, adipose tissue acquires an ability to produce several inflammatory factors, including TNF-α, IL-6, MCP-1, etc., which suppress insulin signaling in this tissue and body-wide (14, 16, 22, 23).

### Liver

Liver insulin resistance manifests through impaired suppression of gluconeogenesis, increased hepatic glucose production, stimulation of *de novo* lipogenesis, and secretion of very-low density lipoproteins (17, 21). Chronic hyperinsulinemia and aging can lead to hepatocyte senescence, steatosis, oxidative stress, and inflammation in this tissue (6, 12, 13, 35). In consequence, there arises a peculiar condition of insulin resistance, where insulin fails to inhibit gluconeogenesis, but stimulates the activity of lipogenic pathways (17, 21).

### Skeletal muscle

Skeletal muscle is the main target of insulin-induced glucose utilization (18, 19, 25). Development of insulin resistance in aged skeletal muscle involves impaired mitochondrial oxidative metabolism, accumulation of lipid intermediates inside muscle fibers, poor capillary perfusion, chronic inflammation, muscle atrophy, and reduction of GLUT4 translocation (13, 26, 27, 29, 30). This results in sarcopenia, poor physical activity, frailty, and loss of metabolic flexibility (26, 27, 30, 31). Intracellular lipid metabolites such as DAG and ceramide inhibit insulin signaling due to their ability to block IR/IRS-AKT signaling cascade (18, 19).

### Pancreatic β-cells

Initial compensation of insulin resistance includes stimulation of insulin secretion. However, continuous metabolic burden, glucolipotoxicity, oxidative damage, endoplasmic reticulum stress, and chronic inflammation gradually decrease the ability of β-cells to synthesize and secrete insulin (3, 4, 14, 17). Aging itself leads to reduced proliferative capacity of β-cells and their sensitivity to stress (3, 4), making them more vulnerable to develop T2D in response to increasing insulin demand.

### Brain

Insulin signaling pathway in brain neurons regulates appetite, learning abilities, synaptic function, neuron survival, neuroinflammation, and metabolic homeostasis (32–35). Aging-dependent insulin resistance in brain may affect brain neuronal glucose uptake, mitochondria functioning, amyloid-beta protein catabolism, tau protein synthesis, and microglia function (32–35). Furthermore, peripheral insulin resistance can cause aging in the brain by exacerbating systemic inflammation, vascular disorders, disruption of the blood-brain barrier, and altered lipid metabolism (32–35).

### Vascular tissue

The impact of insulin resistance on vascular aging cannot be underestimated, as endothelial dysfunction underlies multiple vascular diseases. Under normal conditions, insulin signaling increases nitric oxide synthesis via PI3K-Akt-eNOS signaling, enhancing vasodilation and vascular homeostasis. In contrast, insulin resistance results in defects in this pathway, whereas MAPK-driven effects such as growth and inflammation, endothelin-1 production, and smooth muscle proliferation remain relatively unchanged (36).

A tissue-specific viewpoint allows us to conclude that insulin resistance is not a homogeneous process. Rather, aging affects insulin-sensitive organs specifically, leading to common pathologic mechanisms of insulin resistance, i.e. mitochondrial dysfunction, inflammation, oxidative stress, senescence, ectopic lipid accumulation, and metabolic inflexibility.

## Insights into aging-associated insulin resistance from recent omics studies

Recent insights from transcriptomics, single-cell RNA sequencing, spatial transcriptomics, and integrative bioinformatics have greatly improved our understanding of aging-induced insulin resistance (5–9). In addition to highlighting the roles of aging and metabolic dysfunction caused by adipocyte hypertrophy, the role of adipocyte progenitors, adipose stroma, endothelium, the immune system, and ECM remodeling have also been described (5–8).

Human adipose tissue single-cell and spatial transcriptomic profiling have highlighted the heterogeneity present in both adipose progenitor cells and the stromovascular fraction (6–8). Markers for adipose progenitors/stromovascular cells include PDGFRA, CD55, PI16, SEMA3C, and DPP4 (5–9). Different populations of cells seem to vary with regards to their adipogenic capabilities, inflammatory signaling, ECM remodeling, and relationship with insulin resistance (5–9). The aging process in adipocyte progenitors leads to a reduction in adipose progenitor plasticity and expandability, increased risk of fibrosis, and accumulation of ectopic lipid deposits (5–9).

In addition, several molecular signatures have been described that relate to adipose tissue aging and metabolic dysfunction in human patients (5–9). Genes associated with metabolic pathways, adipogenesis, inflammation, ECM organization, and nutrition sensing are found to be differentially expressed (5–8, 9). The expression of FABP4 and APOE reflects the capacity of lipids to be processed, while ELN may represent ECM remodeling and the reduction in tissue elasticity (7–9). MXD1 represents transcriptional regulation and cell-cycle arrest, and FGF21 is a stress-responsive metabolic hormone involved in lipid oxidation, glucose metabolism, and adaptive metabolic response (9). STAT3 and PCK1 mediate inflammation and glucose/lipid metabolism through interactions between the liver and adipocytes (9).

A number of candidates that regulate aging and insulin resistance of adipose tissue have been suggested recently by means of transcriptomic analysis, single cell RNA sequencing, spatial transcriptomics, and bioinformatics approaches. They include genes and proteins involved in the biology of adipocyte progenitors and stromal cells, complement system, extracellular matrix metabolism, lipid transport, inflammation, nutrition, and cross-talk between liver and adipose tissues. Instead of presenting these markers as being validated for use in clinical practice. **Table 2** highlights some representatives of these molecular mechanisms.

**Table 2:** Selected molecular regulators and biomarkers implicated in aging-related insulin resistance.

Gene/marker	Major tissue/cell source	Main biological role	Relevance to aging-insulin resistance axis
PDGFRA	Adipose progenitor/stromal cells, fibro-adipogenic progenitors	Stromal progenitor identity, adipose remodeling, fibrosis	Marks adipose progenitor populations altered during aging and metabolic dysfunction (5) (8)
CD55	Adipose progenitor cells	Complement regulation, progenitor subset identity	Helps define adipose progenitor subpopulations linked to adipose remodeling and metabolic risk (6)
PI16	Stromal/progenitor cells	Fibroblast/progenitor identity, extracellular matrix regulation	Marker of progenitor populations implicated in tissue remodeling and adipose aging (6, 7)
SEMA3C	Adipose progenitor/stromal cells	Cell guidance, stromal signaling, adipose progenitor identity	Identifies adipose progenitor subsets with altered function in obesity and diabetes (6)
DPP4	Adipose progenitor cells, endothelial/stromal cells	Incretin metabolism, adipose progenitor identity, inflammatory signaling	Links adipose stromal dysfunction with glucose metabolism and insulin resistance (6–8)
FABP4	Adipocytes, macrophages	Fatty acid transport, lipid handling, inflammatory metabolism	Associated with adipocyte dysfunction, ectopic lipid flux, and cardiometabolic risk (7, 8)
APOE	Adipocytes, macrophages, liver, brain	Lipoprotein metabolism, lipid transport, neurodegenerative risk	Connects lipid handling, adipose inflammation, vascular risk, and brain aging (7, 8)
ELN	Stromal cells, extracellular matrix	Elastic fiber structure and tissue elasticity	Reflects extracellular matrix remodeling and age-related loss of tissue elasticity (9)
MXD1	Multiple cell types	Transcriptional repression, cell-cycle control, differentiation	Potential regulator of senescence-associated transcriptional remodeling (9)
FGF21	Liver, adipose tissue, muscle	Metabolic stress hormone, lipid oxidation, insulin sensitivity, anti-inflammatory adaptation	Increases during metabolic stress and aging; may reflect adaptive or compensatory metabolic signaling (9)
STAT3	Immune, adipose, hepatic, endothelial cells	Cytokine signaling, inflammation, JAK-STAT pathway	Links inflammaging, adipose dysfunction, and insulin signaling disruption (16)
PCK1	Liver, adipose tissue	Gluconeogenesis, glyceroneogenesis, lipid-glucose flux	Connects hepatic glucose production and adipocyte lipid handling with insulin resistance (9)

Table of Selected Genes and Markers Discovered in Current Transcriptomic, Single-Cell, Spatial Transcriptomic, and Bioinformatics Research on Adipose Tissue Aging and Metabolism. These biomarkers include those involved in the identity of adipose progenitors, stromal reorganization, regulation of complement activation, lipid metabolism, extracellular matrix architecture, inflammatory signaling, nutrition sensing, and metabolic cross talk between the liver and adipose tissue (5–9), (16).

While these biomolecular markers do offer valuable information about the aging of adipose tissue, they cannot be considered reliable biomarkers of insulin resistance from a clinical perspective. The majority of these genes have been discovered using omics data or in tissue-specific databases and need to be confirmed and replicated for use in clinical practice. Consequently, it would be most appropriate to treat these results as promising mechanistic clues rather than replacing well-known measures of insulin resistance.

Insulin resistance has long been considered to be a major hallmark of metabolism-driven aging; however, the key significance of the current advances in omics is not that they prove insulin resistance as a causative agent of aging per se, but that they improve our understanding of the molecular mechanisms of tissue remodeling during aging and aging-related changes in metabolism. Omics-based single cell and spatial transcriptomic approaches provide an indication that adipose tissue aging involves alterations in the progenitor cells, stroma, endothelial cells, infiltration of immune cells, changes in the extracellular matrix and intercellular communication. These findings make it possible to interpret the mechanism of action of insulin resistance in terms of tissue remodeling, in which impairment of adipose expandability, fibrosis, inflammation and altered progenitor cells function may be associated with systemic metabolic dysfunction.

## Sex and gender differences in aging-associated insulin resistance

Insulin resistance and aging are affected by sex and gender through different types of body fat deposits, sexual hormones actions, muscle mass, inflammatory processes, physical activity, and cardiovascular risk. Women before the period of menopause usually store more subcutaneous fat and demonstrate higher insulin sensitivity than men who frequently accumulate visceral fat and fat ectopic deposition. The period of menopause is considered to be a significant metabolic transformation that takes place due to changes in estrogens and leads to visceral fat formation, inflammation, mitochondrial and endothelial dysfunction, and insulin resistance. The decrease in testosterone concentration in older men can act reciprocally with visceral fat deposition, low muscle mass, and insulin resistance (37, 38).

## Insulin resistance and specific chronic diseases

### Type 2 diabetes and cardiovascular disease

Type 2 diabetes mellitus, stands out as the most clinically overt consequence of chronic insulin resistance. Type 2 diabetes develops after many years of developing progressive insulin resistance and hyperinsulinemia as the body compensates for increasing levels of insulin resistance, ultimately leading to β cell dysfunction and uncontrolled hyperglycemia (3). Nevertheless, cardiovascular disease, which includes heart attack and stroke, poses much higher risks than does diabetes-induced hyperglycemia itself, accounting for the majority of morbidity and mortality in people with type 2 diabetes (3).

Insulin resistance as a risk factor for cardiovascular disease provides some of the most compelling specific disease links discussed in this paper due to its support from epidemiological, mechanistic, and intervention studies. Yet the association is confounded by obesity, hypertension, dyslipidemia, smoking, chronic kidney disease, poor diet, lack of exercise, and diabetes duration. Thus, insulin resistance is better understood within a more holistic cardiometabolic risk network.

### Neurodegenerative diseases: the insulin resistance-dementia connection

The link between type 2 diabetes and increased dementia has been increasingly identified, including the relationship between insulin resistance and Alzheimer’s disease (AD), which is the most prevalent form of dementia (32).

Evidence for the role of insulin resistance in neurodegeneration is biologically plausible, although the proof is not as strong compared with cardiometabolic conditions. Human observational studies may be prone to confounding from vascular disorders, obesity, duration of diabetes, educational status, frailty, systemic inflammation, and medications. Animal studies, as well as mechanistic research, lend support to the role of brain insulin action, neuroinflammation, amyloid metabolism, tau biology, and mitochondria.

### Sarcopenia, frailty, and adipose dysfunction

Insulin resistance can promote sarcopenia and frailty due to muscle protein synthesis impairment, lipotoxicity, oxidative stress, inflammation, mitochondrial dysfunction, anabolic signaling failure, and disrupted adipose-muscle interaction. Nonetheless, sarcopenia and frailty are syndromic conditions determined by aging, inactivity, nutritional factors, co-morbidities, inflammation, hormonal alterations, and neural aspects. Therefore, insulin resistance is just one factor that can exacerbate the functional impairment and not the sole cause of sarcopenia or frailty (27, 31), (**Table 3**).

**Table 3:** Chronic diseases linked to insulin resistance and proposed mechanisms.

Disease/condition	Key insulin resistance–related mechanism	Clinical consequence	Potential intervention
Metabolic syndrome	Impaired insulin signaling, visceral adiposity, dyslipidemia, chronic inflammation	Hypertension, abdominal obesity, hypertriglyceridemia, glucose intolerance	Weight loss, exercise, caloric restriction, whole-food diet, insulin-sensitizing therapy
Type 2 diabetes mellitus	Chronic hyperinsulinemia, progressive β-cell dysfunction, impaired glucose uptake, increased hepatic gluconeogenesis	Hyperglycemia, microvascular disease, cardiovascular risk	Weight reduction, exercise, metformin, GLP-1 receptor agonists, dietary carbohydrate quality improvement
Cardiovascular disease	Endothelial dysfunction, reduced nitric oxide bioavailability, inflammation, oxidative stress, atherogenesis	Coronary artery disease, stroke, arterial stiffness, cardiovascular mortality	Exercise, weight control, lipid/BP control, glucose optimization, anti-inflammatory strategies
Alzheimer’s disease/cognitive decline	Impaired brain insulin signaling, neuroinflammation, mitochondrial dysfunction, amyloid-β accumulation, altered glucose metabolism	Cognitive impairment, dementia progression	Metabolic risk reduction, exercise, insulin-sensitizing strategies, anti-inflammatory and neuroprotective approaches
Sarcopenia/sarcopenic obesity	Impaired muscle insulin signaling, lipid accumulation in skeletal muscle, inflammation, reduced protein synthesis	Loss of muscle mass, weakness, reduced mobility	Resistance training, adequate protein intake, weight optimization, anti-inflammatory strategies
Frailty	Chronic inflammation, oxidative stress, mitochondrial dysfunction, adipose-muscle crosstalk	Functional decline, falls, disability, reduced resilience	Resistance exercise, nutrition optimization, treatment of metabolic syndrome, frailty-focused rehabilitation
Adipose tissue dysfunction	Visceral fat expansion, reduced adiponectin activity, altered adipokines, ectopic lipid deposition	Inflammaging, worsening insulin resistance, metabolic complications	Weight loss, exercise, caloric restriction, improved diet quality
Chronic kidney and liver injury	Lipotoxicity, oxidative stress, endothelial dysfunction, ectopic fat accumulation, inflammation	CKD progression, MASLD/NAFLD, hepatic insulin resistance	Weight loss, metabolic risk control, GLP-1-based therapies, reduced refined carbohydrate intake, exercise

These mechanisms provide a shared biological framework connecting metabolic syndrome, diabetes, cardiovascular disease, neurodegeneration, sarcopenia, frailty, and organ dysfunction.

## Intervention strategies and therapeutic implications

### Lifestyle-based interventions

The main approaches, which are supported by scientific evidence, are caloric restriction and enhanced physical activity. Caloric restriction is known to slow physiological aging and prevent age-associated diseases in large part via lowering levels of insulin in the plasma and promoting insulin clearance by the liver (4). In addition, maintaining healthy body weight via proper nutrition and adequate physical activity should still be the key to preventing chronic diseases and ensuring healthy aging, as epidemiologic studies have found a direct link between rising BMI values and risks of type 2 diabetes, heart disease, and cancers starting already with 20–21 kg/m2 (39).

Exercise, on the other hand, is the most effective strategy because of its ability to induce metabolic integration between muscle tissue, adipose tissue, and the liver, as well as protect against the development of aging-associated diseases. The adaptations induced by either exercise itself or by moderate-to-vigorous physical activity are beneficial in terms of metabolic health in people of all ages, including elderly ones. Exercise involves numerous organs working together to ensure metabolic regulation, and the metabolic challenge imposed by exercise triggers long-term adaptive changes associated with metabolic disease prevention. The exact mechanisms underlying exercise-induced adaptations have not been fully elucidated, but it is clear that exercise promotes better insulin sensitivity, improves mitochondrial function, and decreases systemic inflammation. Resistance training with proper nutrition is thought to offer additional protective effects against sarcopenic obesity and frailty (30).

### Pharmacological and dietary interventions

Insulin secretion-reducing drugs, restoration of normal pulsatile insulin secretory pattern, and enhanced clearance of insulin from the liver are promising means for preventing or delaying the progression of conditions caused by hyperinsulinemia (4). Although the thorough examination of particular drugs falls outside the scope of this paper, popular antidiabetics such as metformin, DPP IV inhibitors, GLP-1 agonists, and thiazolidinediones seem to exert more benefits than mere glucose-lowering effects, being capable of anti-inflammatory action directly targeting the metabolic process (33). It should be noted that certain medications might be used for preventing neurodegenerative diseases (33).

Modifying one’s diet is a relatively simple and cheap way to alleviate insulin resistance and related complications. The reduced consumption of dietary AGEs due to alterations in cooking techniques was shown to improve insulin resistance in individuals suffering from diabetes, polycystic ovary syndrome, being healthy, or having dementia, regardless of the level of physical activity, energy intake, and obesity (24). Following a diet based on whole foods, minimizing consumption of refined carbohydrates, heated products, and increasing the amount of anti-inflammatory components seems to have considerable potential in terms of reducing overall AGE content in the body (25).

The recent development of therapeutic strategies has widened the scope for treating metabolic aging not just by means of glucose-lowering alone. The GLP-1 receptor agonists have the potential to help reduce glucose levels, suppress appetite, lead to weight loss, and possibly reduce cardiovascular risk via their impact on body weight, inflammation, hypertension, vasodilation, and adiposity. In the SELECT study, it was found that semaglutide reduced the incidence of major cardiovascular events in patients with cardiovascular disease along with overweight or obesity, but no diabetes (40). Yet in the elderly population, caution should be exercised regarding the use of GLP-1 receptor agonists.

### Therapeutic targeting of underlying mechanisms

Along with the treatment of insulin resistance itself, the possible therapeutic strategies that could treat the downstream effects like mitochondrial dysfunction, oxidative stress, chronic inflammation, and cellular senescence appear to hold much promise in the future. The interventions targeting mitochondria, such as exercise and caloric restriction, may represent another approach aimed at tackling the core of the problem of insulin resistance and related conditions (13). Also, anti-inflammatory therapy along with traditional diabetes therapy can help to avoid frailty and sarcopenia in aging populations (30).

Recent research provides some promising information about the possible usefulness of the senolytic drugs—drugs that can kill senescent cells—that could help to fight the prosenescent effect of insulin resistance (11). Such drugs as dasatinib and quercetin showed the capacity to prevent the development of hyperinsulinemia-induced senescence in the liver cells (11). Further investigation is needed to explore new therapies that target several aspects of the pathological processes associated with insulin resistance and cellular senescence.

**Figure 3:**
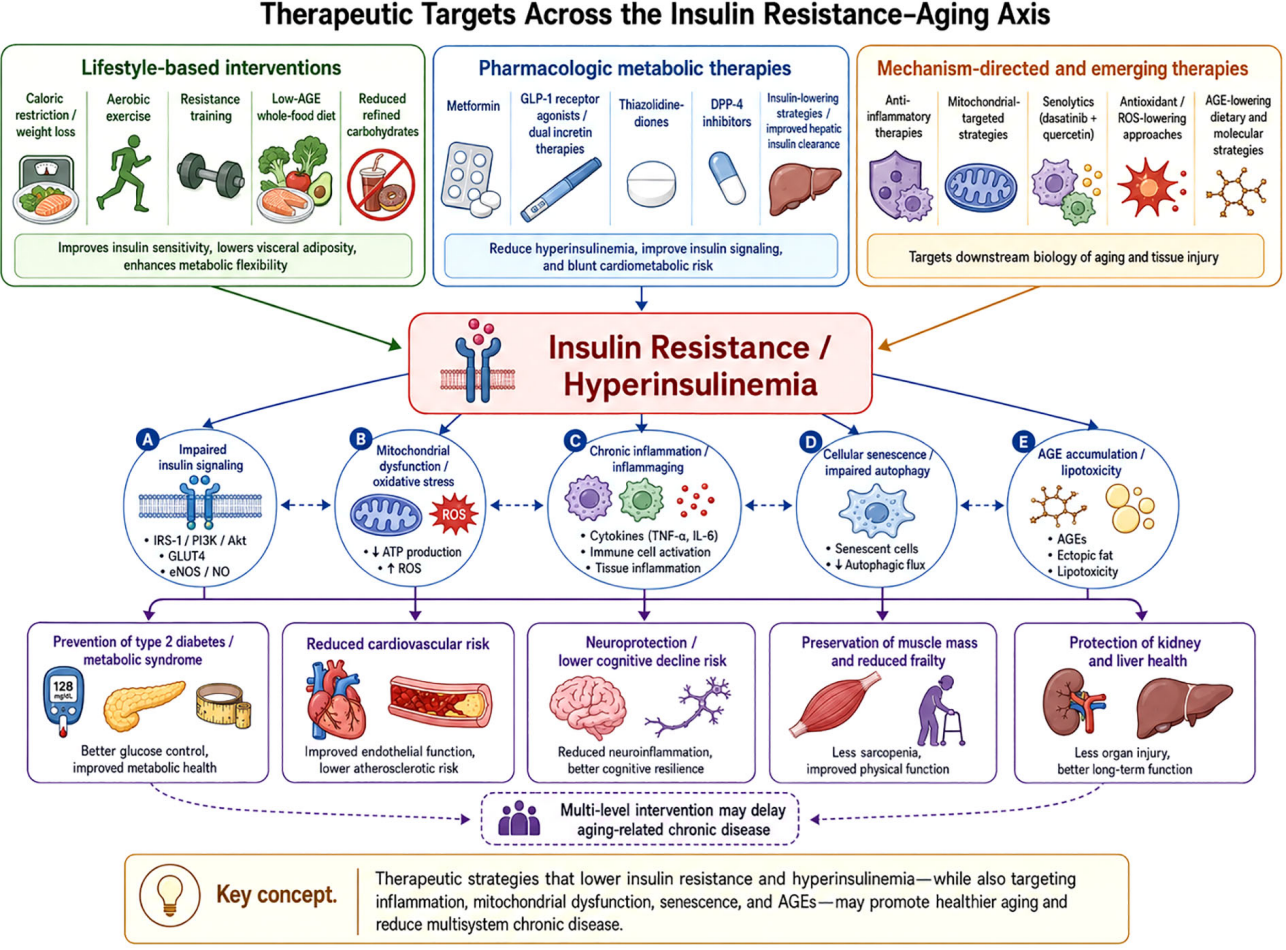
Targeted therapy on the insulin resistance-to-aging pathway. Treatment strategies can address three levels in the insulin resistance aging cascade. First, lifestyle modification therapies, such as reduced calorie consumption, ideal body weight maintenance, aerobic exercise, resistance training, and limited intake of dietary advanced glycation end products, could improve insulin sensitivity, mitochondrial dysfunction, adipose tissue metabolism, and inflammation. Second, metabolic drugs, including metformin, glucagon-like peptide-1 receptor agonists, insulin sensitizers, and cholesterol or blood pressure lowering therapy where appropriate, could mitigate cardiovascular and metabolic risk via the effects of glycemic control, body weight reduction, improved vascular function, decreased inflammation, and lipid metabolism. Third, novel mechanism-based therapies, including senolytic agents, anti-inflammatory therapy, mitochondrial-targeted treatment, and autophagy modulators, are in the investigational phase but could eventually impact downstream processes such as cellular senescence, oxidative stress, inflammation, lipotoxicity, and proteostasis defects. These emerging therapies should be viewed as mechanistic theories unless there is supporting evidence from clinical trials in humans. AGE, advanced glycation end product; GLP-1, glucagon-like peptide-1.

Insulin resistance is known to affect several biological processes at once, and hence treatment modalities should address not only the metabolic causes but also the mechanisms of tissue damage induced by insulin resistance. While lifestyle interventions, such as dietary restrictions, weight reduction, physical activity, and a low-AGE diet based on whole foods, form the basis of treatment, pharmacologic interventions might enhance insulin sensitivity and minimize the hyperinsulinemia and cardiometabolic risk involved; novel treatments based on the understanding of pathophysiological processes are expected to add more value in the future (**Figure 3**).

Whereas the effect of interventions related to lifestyle changes and certain metabolic drugs has been found to have an impact on insulin resistance and cardiovascular risk factors, mechanisms such as senescence, mitochondrial function, and autophagy are still considered experimental in terms of human aging-associated insulin resistance and should therefore not be referred to as clinically established therapy.

Another new therapy associated with the aging and insulin resistance spectrum is senolytics. Senolytic agents include dasatinib, quercetin, and fisetin and have been evaluated for their potential in decreasing the number of senescent cells and senescence-associated inflammation. Preliminary data indicate that senolytics may alleviate age-related dysfunction of adipose tissue metabolism and inflammation (41). Nevertheless, there is still insufficient clinical data on the effects of senolytics, and these agents cannot be regarded as proven treatment for insulin resistance and other diseases of aging. Future clinical trials will help define their effectiveness in improving insulin resistance and other parameters related to age-related diseases.

## Discussion and future directions

The current findings indicate that insulin resistance, as well as the resultant compensatory hyperinsulinemia, could indeed serve as the biological mechanisms behind the association between aging and many age-related non-communicable diseases. Nonetheless, the connection must be considered not as a linear one but a complex bidirectional network. Aging increases insulin resistance via senescence, mitochondrial dysfunctions, disruption of proteostasis, inflammation, and altered nutrient sensing, while insulin resistance may speed up aging phenotypes via metabolic stresses, oxidative damage, inflammation, defective autophagy, and tissue remodeling (5–8), (9), (16). The review provides strong support for the notion that insulin resistance and compensation are key elements of the biological pathway between metabolic dysfunction, aging, and age-related non-communicable diseases. Nevertheless, the association should not be seen as a purely linear and causative one. Ageing might lead to insulin resistance by increasing cellular senescence, mitochondrial dysfunction, proteostasis malfunction, chronic inflammation, aberrant nutrient sensing, epigenetic reprogramming, fat tissue dysfunction, and decreased physical activity. On the other hand, insulin resistance could increase age-induced tissue dysfunction through metabolic disturbance, oxidative damage, endothelial dysfunction, lipotoxicity, autophagy malfunction, and inflammation.

Evidence strength varies in terms of different disease domains. The association between insulin resistance and cardiometabolic outcomes is backed up by better epidemiologic, mechanistic, and intervention evidence; whereas the connection with neurodegeneration, sarcopenia, frailty, and omics-based tissues is less homogeneous and mostly associative. Thus, insulin resistance should be considered as a context-dependent amplifier of age-related tissue malfunction rather than the single primary cause of aging.

Insights from recent omics research allow for greater biological understanding by showing cell and tissue-specific changes in adipocyte progenitors, stromal cells, immune cells, endothelial cells, ECM remodeling, lipid metabolism, and inflammatory response. The results imply that insulin resistance associated with aging involves tissue remodeling and inter-cell communication and not just mature adipocytes’ dysfunction. Nevertheless, most of the insights from omics research are still largely hypothesis-generating and need longitudinal confirmation prior to translation to clinic.

Therapeutically, lifestyle modification, weight loss, physical activity and some metabolic therapies have better evidence in terms of improving insulin resistance and cardiometabolic risk. Meanwhile, therapies addressing cellular senescence, mitochondria dysfunction, autophagy malfunction, or certain omics-derived pathways are all experimental in humans at this point. Further research should investigate whether improvement in insulin resistance leads to better functional outcomes like frailty, sarcopenia, cognitive decline, heart attacks, inflammatory load, mitochondria health, and quality of life.

Omics approaches have been able to add another dimension to this hypothesis in establishing that insulin resistance associated with aging is caused by the remodeling of the adipose tissue involving multiple cells. Changes in adipose progenitor cells, stroma cells, infiltration of immune cells, alterations in endothelium, remodeling of the extracellular matrix, and genes like PDGFRA, CD55, PI16, SEMA3C, DPP4, FABP4, APOE, ELN, MXD1, FGF21, STAT3, and PCK1 indicate that metabolic aging cannot be attributed just to mature adipocytes alone (5–8), (9).

This paradigm shift implies major shifts in therapy regarding insulin resistance. If hyperinsulinemia, which is believed to induce insulin resistance, is recognized as a causative factor rather than a compensatory phenomenon for insulin resistance, this means that insulin resistance, in turn, can be successfully targeted in the early stages of life before chronic disease onset. The treatment would mean preventing the occurrence of all age-related chronic diseases at once instead of having to treat each disease separately as a single entity.

Despite numerous discoveries already made in relation to insulin resistance, many knowledge gaps still exist. First of all, more research is required in order to understand the specifics of insulin resistance in certain tissues and ages, identify specific risk factors that make someone vulnerable, and find more effective interventions to address insulin resistance. Furthermore, large-scale clinical trials are necessary to confirm the effectiveness of different interventions aimed at the prevention of age-related diseases through lowering or avoiding insulin resistance.

### Directions for future research

Future work needs to move away from the systemic nature of insulin resistance toward defining insulin resistance phenotypes specific to skeletal muscle, liver, fat tissue, brain, vascular tissue, and pancreatic β-cells. Longitudinal studies are required to clarify whether insulin resistance is upstream of aging phenotypes or if the aging process drives tissue dysfunction that leads to insulin resistance. Biomarkers for insulin resistance-induced aging need to be determined using multi-omic, imaging, metabolomics, proteomics, and single-cell technologies.

Future clinical trials are required to evaluate whether strategies to lower or sensitize to insulin alter aging-related outcomes in older people. Trials will need to look at more than just glucose metabolism; other outcomes that could be studied include frailty, sarcopenia, cognitive decline, cardiovascular events, function, inflammation, mitochondrial health, and quality of life. Interventions like exercise, caloric restriction, GLP-1 receptor agonists, senolytic drugs, anti-inflammatory drugs, and mitochondrial-targeted therapies need to be studied with respect to their differential impact in individuals of differing sex, menopausal status, body habitus, and preexisting frailty. Lastly, hyperinsulinemia, insulin resistance, β-cell dysfunction, and biological aging need to be studied longitudinally in humans.

## Limitations of current evidence

There are a number of limitations in existing research which need to be addressed. First, much of the existing research linking insulin resistance with diseases associated with aging is observational, and thus does not prove causation. Second, there are problems in defining insulin resistance through the use of systemic biomarkers like fasting insulin and HOMA-IR. Third, there are a number of differences between various groups in terms of age range, metabolic phenotypes, sex, ethnicity, adiposity, medications and presence of other diseases that prevent comparability across studies. Fourth, although mechanisms discovered in animals and cells might provide insight into human aging, there are many differences since the aging of humans takes years and is influenced by environmental factors, lifestyles, epigenetic changes and physiological deterioration. Finally, while omics research has been helpful in discovering possible genes and cell states, they still need to be validated in order to become clinically relevant biomarkers or targets for therapy (5–8), (9), (16).

## Limitations of the review

There are multiple limitations associated with this review. Firstly, it should be noted that the current research is a narrative review and hence lacks risk-of-bias assessment, quantitative synthesis, and certainty assessment. Secondly, while a structured methodology for searching articles was employed, there could be some selection bias involved in this process, as all decisions on selecting articles were made based on their applicability to the review aims and questions. Thirdly, most of the links between insulin resistance and diseases caused by aging are based on observational data and thus cannot imply causality. Fourthly, a number of studies employ systemic markers such as fasting insulin levels, HOMA-IR index, or other surrogate metabolic indexes, which do not necessarily reflect insulin resistance at a cellular level. Fifthly, mechanistic studies conducted using animal models, cell cultures, and certain tissues of the body cannot be easily translated into real-life conditions of human aging. Sixthly, while the hyperinsulinemia-first model is often accepted, one still needs to consider alternative mechanisms, such as inflammation-driven insulin resistance, mitochondrial aging, primary failure of pancreatic beta cells, and epigenetic reprogramming.

## Critical appraisal

There is a great deal of variability in the level of evidence associating insulin resistance with age-associated disease depending on the clinical field. The strongest evidence exists to associate insulin resistance with cardiometabolic disease, including metabolic syndrome, type 2 diabetes, cardiovascular disease, and fatty liver disease, as there is general agreement between epidemiology, biology, and intervention. However, for conditions such as neurodegenerative disease, sarcopenia, frailty, senescence, and cellular aging, evidence is weaker and more dependent on observational research, animal models, *in vitro* studies, omics studies, and biological plausibility. Thus, insulin resistance can be thought of as a condition-specific metabolic amplifier operating within age-disease feedback loops. It is not an overarching cause of biological aging. Further long-term human studies of the effects of improved insulin sensitivity on age-associated clinical endpoints are needed.

## Conclusion

Insulin resistance is strongly connected with biological aging and age-related non-communicable chronic diseases by means of a mutual metabolic, inflammatory, mitochondrial, endothelial, senescence-related, and tissue remodeling pathway. Nevertheless, one has to bear in mind that such connections may not be regarded as unambiguously causal. There is ample proof for cardiometabolic conditions, which include metabolic syndrome, type 2 diabetes, cardiovascular disease, and fatty liver disease. The association between insulin resistance and neurodegeneration, sarcopenia, frailty, and tissue-specific aging is weaker and still has an experimental character. Insulin resistance can be regarded as a metabolic amplifier in the context of biological aging and chronic disease.
